# Aberrant right subclavian artery and calcified aneurysm of kommerell's diverticulum: an alternative approach

**DOI:** 10.1186/1749-8090-3-43

**Published:** 2008-07-09

**Authors:** Jose Rubio J Alvarez, Sierra JL Quiroga, Adrio B Nazar, Martinez JM Comendador, Garcia J Carro

**Affiliations:** 1 Department of Cardiovascular Surgery, University hospital Santiago de Compostela, Spain

## Abstract

We report a 72 year-old man with dysphagia and dizziness. Aortography and Computed tomographic scans revealed the aberrant right subclavian artery arising from a calcified aneurysm of the Kommerell's diverticulum and bilateral carotid artery disease with atherosclerotic narrowing. Surgical relief was accomplished by excluding the aneurysm from circulation through the aortic arch and a 10 mm graft was interposed between the aberrant artery and the ascending aorta.

## Background

Aberrant right subclavian artery (ARSA) with Kommerell's diverticulum, is a rare congenital anomaly of the aortic arch found at postmorten examination with a frequency of 0,5% [1].

In this lesion the right subclavian artery arises as the fourth branch of the aorta distal to the origin of the left subclavian artery, then comes to the right arm posterior to the esophagous. This anomaly occurs as a result of abnormal regression of the fourth aortic arch and persistence of patency of the right eight dorsal aortic segment [2]. An aortic diverticulum is found at the site of origin of the atretic arch and this diverticulum is termed the Kommerell's diverticulum [3]. A patient with an ARSA and calcified aneurysm of Kommerell's diverticulum is described in whon a new surgical approach was taken.

## Case report

A 72 year-old man who had a 10 – year history of hypertension, noninsulin-dependent diabetes, coronary artery disease (right coronary stent) and dizziness was referred to our hospital for severe dysphagia.

**Figure 1:**
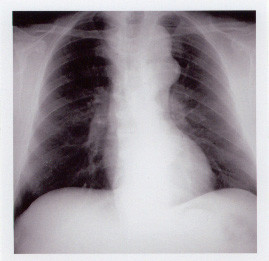
Chest X-ray film showing a mass in the upper mediastinum.

**Figure 2:**
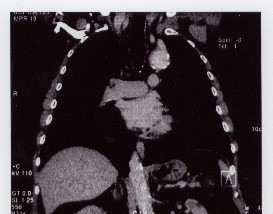
Computed Tomographic Scans revealed a large and calcified aneurysm of Kommerell's diverticulun.

Chest X-ray films showed a mass in the upper mediastinum (Fig 1). Computed tomographic scans (Fig 2) revealed a calcified aneurysm (5 cm) of an aberrant right subclavian artery and an abdominal aortic aneurysm (4 cm). Aortography demonstrated that the right and left carotid arteries and the left subclavian artery arose from the aortic arch in that order. The ARSA arose from the aneurysm of the Kommerell's diverticulum in the descending aorta.

Bilateral carotid artery disease with proximal atherosclerotic narrowing was present. Elective surgery was performed through a midline sternotomy using cardiopulmonary bypass (33°) (CPB) and cold antegrade blood cardioplegic cardiac arrest. CPB was established with cannulation to the right femoral artery, the right common and the left common carotid arteries and right atrium. The left heart was vented through the right superior pulmonary vein.

The ascending aorta was clamped and the heart was arrested, selective cerebral perfusion was started and the two carotid arteries and the left subclavian artery were clamped. The ARSA was ligated distal to the aneurysm and behind the ascending aorta. The perfusion through the femoral artery was stopped, the end of the aortic arch was incised and an arterial occlusion catheter was inserted directly into the descending aorta and inflated, then we began the distal perfusion. The origin of the ARSA was posterior and was closed with a Dacron patch using horizontal mattress sutures with pledgets, thus excluding the aneurysm from the circulation.

A right subclavian – ascending aorta bypass was performed using an 10 mm collagen-impregnated polyester graft. Postoperatively, the patient was neurologically intact and hemodynamically stable but needed mechanical ventilation for 10 days. The patient was discharged in good condition 22 days after operation and in the subsequent 3 years has remained well.

## Discussion

Aberrant right subclavian artery occurs as a result of abnormal regression of the fourth aortic arch and persistence of patency of the right eight dorsal aortic segment [2]. This is a rare congenital anomaly of the aortic arch found at postmorten examination with a frequency of 0,5% [1]. Sixty percent of patients with ARSA have Kommerell's diverticulum [4] and aneurysm of the diverticulum have been observed in 3–8% of these patients [4].

Most patients with ARSA are asymptomatic, but during adulthood 5% of patients with this anomaly have symptoms because the development of atherosclerosis and dilatation of Kommerell's diverticulum results in compression of the esophagus or the trachea [5].

Because of the rareness of the ARSA with aneurysm of Kommerell's diverticulum surgical indications and stategies are still unclear, however rupture and dissection of these aneurysms have been reported. In a review of 32 patients, Austin [6] reported a rate of rupture of 19%. Cina [7] reported a rate of rupture of 53% and Kouchoukos [8] reported a rate of dissection of 20%. Based upon these reported series, early surgical intervention appears appropriate.

Cina and Colls [7] recommended surgical treatment for aneurysms 3 cm or greater and in Ota experience [9] your primary indication was that the diameter of aneurysm was more than 50 mm. Surgical standards are the resection or exclusion of the aneurysm and the reconstruction of the aberrant subclavian artery. The choice of surgical approach depends on the anatomic details, the urgency of the surgery and the surgeon's experience.

In the bibliography, the majority of procedures were two-staged operations divided into the aneurysm of Kommerell's diverticulum and the aberrant subclavian artery through separate approaches [6,9]. Kouchoukos et collbs [8] recommended selective carotid-subclavian artery bypass, thoracotomy and cardiopulmonary bypass as the optimal method of management of patients with this anomaly, but we decided to perform a one-stage operation through median sternotomy and under cardiopulmonary bypass because our patient had bilateral carotid artery disease with atherosclerotic narrowing, calcified aneurysm of Kommmerell's diverticulum and abdominal aneurysm.

In patients with bilateral carotid disease, as in the present case, the most straightforward option would be an ascending aorta – subclavian bypass. On the other hand, we chose the " no touch technique " because the aneurysm was calcified, excluding the aneurysm from circulation through the aorta. Transluminal placed stent-grafts offer an alternative approach to the standard surgical treatment [10] and this is less invasive but we believed that it is associated with higher rates of morbidity when severe atherosclerotic disease of the abdominal aorta is present, as in our case.

## Authors' contributions

RJ and GJ Surgeons. SJL and AB Surgeons in charge of postoperative period. MJM Compiled the patients' records.
